# Personalized AI-Driven Real-Time Models to Predict Stress-Induced Blood Pressure Spikes Using Wearable Devices: Proposal for a Prospective Cohort Study

**DOI:** 10.2196/55615

**Published:** 2024-03-25

**Authors:** Ali Kargarandehkordi, Christopher Slade, Peter Washington

**Affiliations:** 1 Department of Information and Computer Sciences University of Hawaii at Manoa Honolulu, HI United States

**Keywords:** stress, hypertension, precision health, personalized artificial intelligence, wearables, ecological momentary assessments, passive sensing, mobile phone

## Abstract

**Background:**

Referred to as the “silent killer,” elevated blood pressure (BP) often goes unnoticed due to the absence of apparent symptoms, resulting in cumulative harm over time. Chronic stress has been consistently linked to increased BP. Prior studies have found that elevated BP often arises due to a stressful lifestyle, although the effect of exact stressors varies drastically between individuals. The heterogeneous nature of both the stress and BP response to a multitude of lifestyle decisions can make it difficult if not impossible to pinpoint the most deleterious behaviors using the traditional mechanism of clinical interviews.

**Objective:**

The aim of this study is to leverage machine learning (ML) algorithms for real-time predictions of stress-induced BP spikes using consumer wearable devices such as Fitbit, providing actionable insights to both patients and clinicians to improve diagnostics and enable proactive health monitoring. This study also seeks to address the significant challenges in identifying specific deleterious behaviors associated with stress-induced hypertension through the development of personalized artificial intelligence models for individual patients, departing from the conventional approach of using generalized models.

**Methods:**

The study proposes the development of ML algorithms to analyze biosignals obtained from these wearable devices, aiming to make real-time predictions about BP spikes. Given the longitudinal nature of the data set comprising time-series data from wearables (eg, Fitbit) and corresponding time-stamped labels representing stress levels from Ecological Momentary Assessment reports, the adoption of self-supervised learning for pretraining the network and using transformer models for fine-tuning the model on a personalized prediction task is proposed. Transformer models, with their self-attention mechanisms, dynamically weigh the importance of different time steps, enabling the model to focus on relevant temporal features and dependencies, facilitating accurate prediction.

**Results:**

Supported as a pilot project from the Robert C Perry Fund of the Hawaii Community Foundation, the study team has developed the core study app, CardioMate. CardioMate not only reminds participants to initiate BP readings using an Omron HeartGuide wearable monitor but also prompts them multiple times a day to report stress levels. Additionally, it collects other useful information including medications, environmental conditions, and daily interactions. Through the app’s messaging system, efficient contact and interaction between users and study admins ensure smooth progress.

**Conclusions:**

Personalized ML when applied to biosignals offers the potential for real-time digital health interventions for chronic stress and its symptoms. The project’s clinical use for Hawaiians with stress-induced high BP combined with its methodological innovation of personalized artificial intelligence models highlights its significance in advancing health care interventions. Through iterative refinement and optimization, the aim is to develop a personalized deep-learning framework capable of accurately predicting stress-induced BP spikes, thereby promoting individual well-being and health outcomes.

**International Registered Report Identifier (IRRID):**

DERR1-10.2196/55615

## Introduction

### How This Research Benefits the People of Hawaii

According to the Department of Health Chronic Disease Prevention and Health Promotion Division, 1 in every 3 adults in Hawaii has been diagnosed with hypertension [1]. Mortality rates associated with heart disease are particularly high for Native Hawaiian and Other Pacific Islander populations, leading to 628 deaths per 100,000 residents as opposed to 154 deaths per 100,000 residents among Asian residents and 167 deaths per 100,000 among White residents in Hawaii [1].

A recent study conducted by researchers at the John A Burns School of Medicine found that Native Hawaiian and Other Pacific Islander individuals under a physician’s care for hypertension experienced an 18.3 point drop in systolic blood pressure (BP) on average when participating in a 12-week hula program [2,3]. This study provides strong evidence that stress-reducing interventions can reduce hypertension in Native Hawaiian individuals. We hope to build upon this foundational research by leveraging consumer devices (ie, Fitbit) to detect moments of high stress and to provide just-in-time interventions that are culturally grounded. The first step of this long-term research plan is to develop the artificial intelligence (AI) that will power the digital intervention, and that first step is the focus of this grant proposal.

### Clinical and Unmet Needs

Hypertension is an indirect cause of hundreds of thousands of annual deaths in the United States alone [4]. Known as the “silent killer”[5], elevated BP often remains unnoticed by affected individuals due to a lack of perceptible symptoms, resulting in accumulated harm over the years. While several causes of hypertension are related to an underlying health condition such as kidney disease, diabetes, sleep apnea, or hormone problems [6]; health condition; and medications combined only account for roughly 1 in 20 cases [7]. Chronic stress has been repeatedly documented to increase BP [8-10].

Prior studies have found that elevated BP often arises due to a stressful lifestyle, although the effect of exact stressors varies drastically between individuals. Due to the heterogeneous nature of both the stress and BP response to a multitude of lifestyle decisions, it can be difficult if not impossible to pinpoint the most deleterious behaviors in a personalized manner using the traditional mechanism of clinical interviews. Passive sensing technologies deployed on consumer devices have the potential to disrupt this status quo in a positive manner. By continuously monitoring a patient’s lifestyle in naturalistic settings, digital technologies can provide clinicians and patients alike with actionable insights into their health trends with fine-grained precision.

We are interested in the use of wearable technologies to sense cardiovascular signals, as they are noninvasive and are already widely adopted. We will develop machine learning (ML) algorithms that analyze these biosignals to make real-time predictions about BP spikes. The resulting predictions could be used to alert, in real time, patients about unintentionally adverse behaviors as well as clinicians about the frequency of such behaviors. There is a critical opportunity and need to improve diagnostics for repeat health events to enable clinicians to monitor their patients and forecast future issues.

### Innovation

There are countless situations in health care and biomedicine where vast amounts of unlabeled data are collected from a single patient [11]. Annotations for the event of interest are frequently sparsely dispersed. The development of predictive supervised ML models is infeasible in such circumstances, as classical approaches cannot handle the complexity of the data and modern deep learning approaches require vast amounts of data [12]. For example, continuous readings from continuously worn glucose monitors can provide enough input data to train a model to make a prediction about patient energy based on glucose, but it is impracticable to require users to log their perceived energy at the same sampling frequency as a wearable device. Similar situations arise from data collected by consumer wearable health devices (eg, smart watches), smartphones, and other devices that measure biological signals.

**Figure 1:**
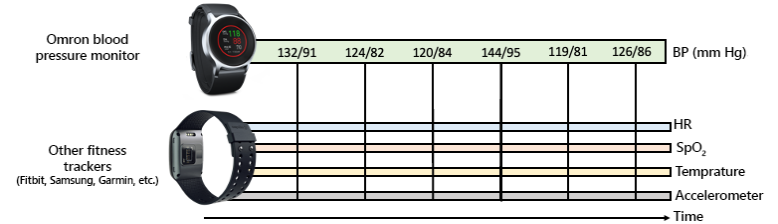
We will collect wearable biosignals from a Fitbit and use them to predict BP as measured by an Omron HeartGuide wearable BP monitor. We will use personalized self-supervised learning to enable the prediction of BP using minimal samples from the end user. BP: blood pressure; HR: heart rate.

To support AI development in these situations where vast longitudinal data are collected with minimal human-provided annotations, we propose the development of personalized ML models that are trained solely on an individual’s unlabeled data to learn feature representations that are specific to their baseline temporal dynamics. We will train these models with a novel data set of Fitbit biosignals and corresponding BP readings (Figure 1). We are creating a novel method and framework, which has never been explored in health care, consisting of pretraining neural networks to learn the temporal dynamics of a patient’s biosignals. This method will enable powerful, deep networks to be trained using relatively small data sets, which would not be possible without the self-supervised approach proposed here. From a usability standpoint, patients will only be required to provide tens of annotations tens of times to get a personalized predictive model.

While we propose to apply this new technological innovation toward the prediction of cardiac signals, multimodal time-series personalization can be applied to a variety of other biology and health problems where (1) multiple signals are emitted, (2) the baseline signal patterns are specific to each individual or organism, and (3) it is infeasible to acquire the vast amounts of labels required to train a supervised deep learning model from scratch. Examples of future apps of the proposed methodology include predictions stemming from nanopore signal data or multielectrode neuronal recordings. This method has the potential to dramatically advance the field of precision health care by enabling reliable ML predictions from massive but mostly unlabeled data sets which are trained in a self-supervised manner on data from a single user.

While this novel methodology could be applied to myriad domains within health and biology, a natural application is the prediction of cardiac events from wearable biosignals data. We will focus on high BP.

### Dissemination Plan

We plan to disseminate our research findings through a combination of (1) research publications in journals, (2) presentations at conferences, (3) as preliminary data for an National Institutes of Health (NIH) R01 application, and (4) as the basis of community-based participatory design sessions where we iteratively develop a culturally informed digital intervention using the AI created in this project. Target journals for submission include Nature Digital Medicine, Science Translational Medicine, Institute of Electrical and Electronics Engineers (IEEE) Transactions on Affective Computing, PLoS Digital Medicine, and Cell patterns. Target conferences include the American Medical Informatics Association (AMIA) Annual Symposium, the Pacific Symposium on Biocomputing (PSB), and the Conference for Computer-Human Interaction (CHI). There are several notices of special interest posted by the NIH that would support a large R01 grant application using the preliminary data from this work.

### Specific Aims

We propose the following specific aims: (1) aim 1: create a novel data set of wearable sensor data and corresponding BP measurements, (2) aim 2: develop a personalized self-supervised pretraining procedure for time-series data using both contrastive learning and masked predictions, and (3) aim 3: develop a novel personalized pretraining procedure which exploits the multimodal nature of wearable time series-data.

## Methods

### Recruitment

We will recruit 40 carefully selected participants with diagnosed hypertension and self-reported stressful lifestyles to each participate in a 4-week remote data collection period. Each participant will wear an Omron HeartGuide BP wearable device and a Fitbit Sense 2 wearable watch during all waking hours for at least 15 hours each day. Apart from wearing the devices and periodically syncing the data to the cloud, participants will be asked to follow their normal routine for the duration of the study.

We will recruit adults aged 30 to 70 years in the state of Hawaii who have been diagnosed with hypertension and self-identify as living a high-stress lifestyle. Given the diversity of the population of Hawaii [13], we aim for the following demographic composition of our participants: 23% White, 37% Asian, 11% Native Hawaiian or Pacific Islander, 7% Black or African American, and 22% of 2 or more races. Approximately 9.5% of the recruited population will have Hispanic or Latino ethnicity.

PW has a network of clinical collaborators at the John A Burns School of Medicine at the University of Hawaii at Mānoa who also practice at local medical centers such as Queen’s Medical Center and Kaiser Permanente’s branch in Hawaii. We will recruit using the following sources: (1) direct recruitment from the Hawaii Pacific Health Center, which the collaborators at the Department of Psychiatry at the University of Hawaii are affiliated with and where they practice clinically; (2) via flyers and emails at the clinics which the Department of Psychiatry at the University of Hawaii regularly provides inpatient and outpatient psychiatric services and consultation at, including The Queen’s Medical Center, Kapiʻolani Medical Center for Women and Children, and Hawaii State Hospital Community mental health centers on Hawaii Island, Molokaʻi, Maui, Kauaʻi, and Lānaʻi; (3) advertisements posted on the University of Hawaii campus and in public settings in Honolulu; and (4) targeted advertisements posted to social media websites. We will work with Anthony Guerrero, the chair of the Department of Psychiatry at the University of Hawaii, to ensure that the recruitment strategies and advertisement of the research program translate across cultures and to ensure effective recruitment as well as diverse and representative data.

We will exclude participants younger than 30 years and older than 70 years. We will require all potential participants to remotely complete the Perceived Stress Scale (PSS), a 10-item scale that is the most widely used psychological instrument for measuring the perception of stress [14]. We will exclude participants whose PSS score does not exceed 1 SD above the mean for at least one of their demographic brackets (age, gender, or race) as reported by Cohen et al [14]. We will also ask participants to self-report their BP. We will also exclude participants who do not own a smartphone with continuous network connectivity. During the in-person study intake, we will measure the BP of potential study participants 3 times. We will exclude participants whose BP does not exceed 130/80 mm Hg for at least one of the measurements, as 130/80 mm Hg is the minimum cutoff for stage 1 hypertension.

### Data Collection and Storage

We will leverage the existing application programming interface (API) provided by both Omron and Fitbit to record the user’s wearable sensor readings and upload the data to the cloud. Omron’s health care API offers access to time-stamped BP readings as well as activity and sleep approximations. The Fitbit API provides access to sensor readings of heart rate (HR), gyroscope, accelerometer, breathing rate, blood oxygen levels (SpO_2_), and skin temperature sensor readings. The data will be managed on each participant’s smartphone devices through a mobile app, implemented for both iOS (Apple Inc) and Android, that we will develop. The study team will install the app on the user’s smartphone and configure the Omron and Fitbit devices during study onboarding. The smartphone app will periodically trigger a notification reminding the participant to (1) measure their BP with the Omron wearable, (2) sync the Omron and Fitbit data to the app, and (3) connect to a network while the study app is open to allow the data to be uploaded to a centralized server.

We will store the curated data from each participant on a centralized server hosted on Amazon Web Services (AWS). Because Fitbit is owned by Google, participants' Fitbit data will be uploaded directly to Google's cloud servers, which use the same level of security as other Google products such as Gmail. Access to each participant's Fitbit data on Google's cloud servers is implemented through OAuth, which provides clients with secure delegated access to server resources on behalf of a resource owner (ie, the participants of this study). This mechanism is used by companies such as Amazon (Amazon.com, Inc), Google (Alphabet Inc), Facebook (Meta Platforms, Inc), Microsoft Corporation, and X (X Corp) to permit users to share information about their accounts with third-party applications or websites. In this case, the “third party” is the study team. The Fitbit data and BP readings will be preprocessed on an Elastic Cloud Compute instance on AWS, which is HIPAA (Health Insurance Portability and Accountability Act)-compliant. The Elastic Cloud Compute instance will store the data onto respective database tables using DynamoDB (Amazon.com). Each table will have columns for the child ID and the time-stamp. We will encrypt all server-side data and require secret access keys for data access. DynamoDB tables are automatically encrypted on the server side. To add an additional layer of security, we will implement client-side encryption on the mobile app, ensuring encrypted data transmission over HTTPS connection to move BP data between the devices and AWS. Data access will require a secret access key provided by the AWS administrators to any data analysis team. The data will not be accessible without this key. For further security, we will anonymize all user data on the server side by removing all protected health information from the DynamoDB tables.

**Figure 2:**
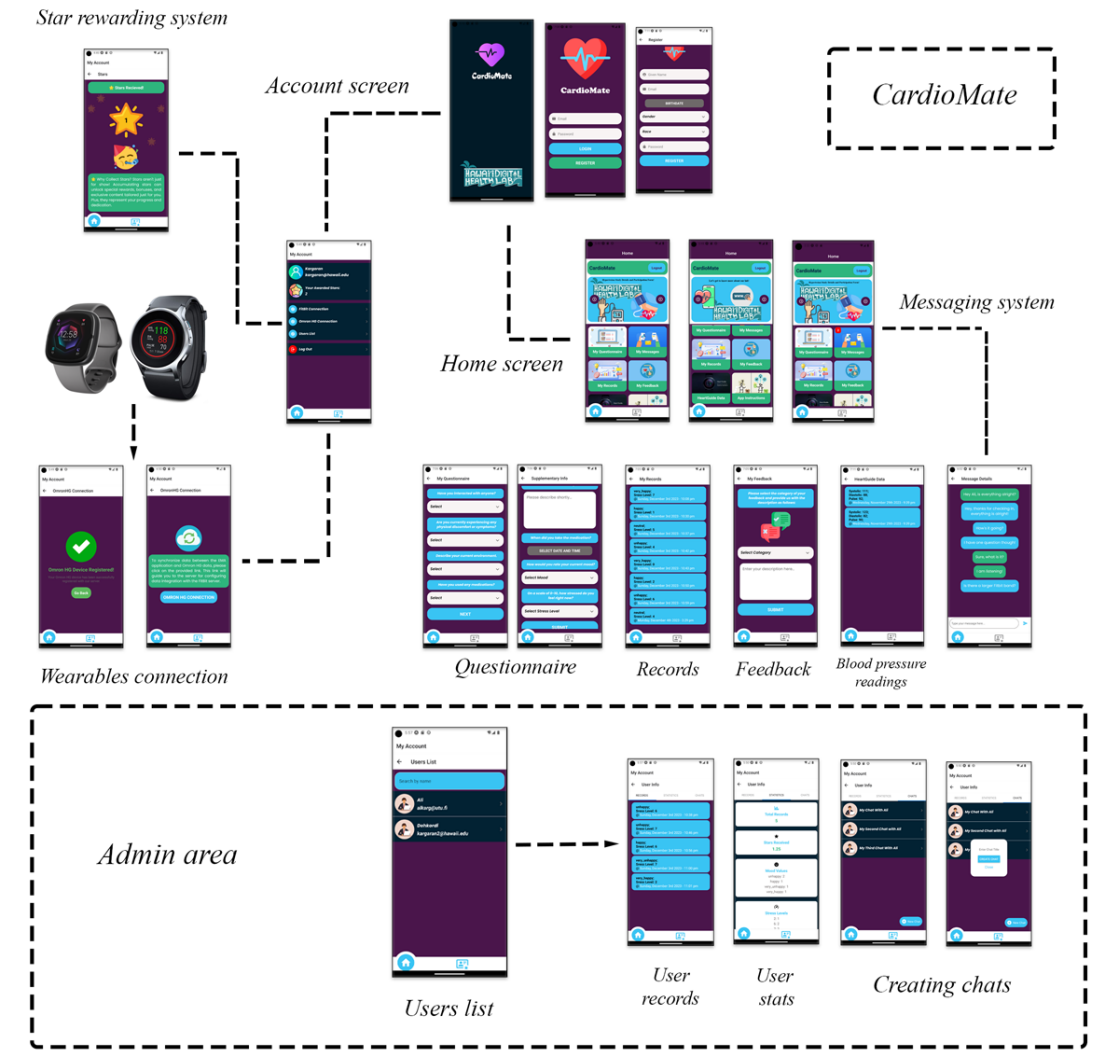
Workflow of the CardioMate app, comprising account and home screens with user details, study rewards, wearable device integration, and an administrative section for study managers.

We intend to release the curated data (Figure 2) as a publicly available data set for use in the evaluation of multimodal time-series ML models. Such data sets exist for activity and emotion recognition from wearable data, but the prediction of BP from these measurements will be a challenging task that other researchers can attempt with the release of our data set. This will be the first publicly available data set that includes at-home BP measurements alongside wearable sensors such as HR, SpO_2_, and accelerometer readings. This fully anonymized data set will only be released to researchers who sign a Data Use Agreement, which will be approved by the University of Hawaii Data Governance Office.

The app comprises 2 primary screens, account and home. The account screen features user details, a star reward system for active participation in the study, and options to link 2 wearable devices (Fitbit and Omron Heartguide) for data synchronization with our secure and encrypted database. The home screen is divided into 6 sections, including questionnaires, messages, feedback, records, BP readings, and app instructions. Additionally, the CardioMate app includes an administrative area for study managers to view participant statistics and initiate personalized chats, complete with alarm and notification functions.

### Feasibility

The most difficult aspect of this aim will be maintaining participant engagement throughout the 4-week study period. The graduate research assistant funded by this project will dedicate some time each day toward running the study and interfacing with participants. We expect participants to open the smartphone app to sync and upload their data on a daily basis, which is a 1-minute time commitment per day.

While we expect no trouble recruiting 40 subjects for participants, we expect some participants to drop off during the study. Since we will have enough devices for 5 concurrent subjects, it will take 8 months to collect all data if no participants drop off. Our study timeline allocates 6 additional months of make-up time to collect data from new participants, accounting for >50% drop-off rate. Given the remote nature of the data collection procedures, we expect some participants to drop off from the study prematurely or to not comply with the study processes. We will therefore remotely monitor the upload progress and send an automated text and email notification to the participant if the data are not uploaded in a timely manner. If 3 consecutive days of participant noncompliance are detected, we will contact the participant for a device return.

### Ethical Considerations

Under an expedited review procedure, this research project was approved on April 26, 2023, by the University of Hawaii Institutional Review Board (UHMUIF_2023-00130). The application qualified for expedited review under CFR 46.110 and 21 CFR 56.110, categories 1b, 4a, 4d, and 6. The informed consent process for this human subject research study involves participants completing an interview session where they receive comprehensive information about the research, including its purpose, procedures, and potential risks and benefits. Participants are assured of the voluntary nature of their participation and their right to refuse or withdraw at any time without penalty. For secondary analyses of research data, it is clarified that the original informed consent allows for such analyses without additional consent, as approved by the institutional review board. Privacy and confidentiality protections are emphasized, with participant data anonymized and stored securely on HIPAA-compliant servers. Compensation for participation includes US $135 upon completion along with an additional US $15 for certain eligibility interview tasks, reflecting the time and effort required from participants while respecting the ethical standards. The consent form ensures that no identification of individual participants or users is possible in any images or supplementary materials without explicit consent, with researchers providing relevant consent forms or written communications to uphold participant privacy and consent.

### AI Model Training

Self-supervised learning (SSL) is usually used to pretrain an entire data set with no explicit labeling by humans to guide the supervision task. We propose to redesign the SSL paradigm toward the task of model personalization. By pretraining a model only on the vast amounts of data curated from a single individual, the weights of the neural network will learn to make predictions using the inherent structure of each participant’s biosignals. This is essential because the baseline HR, SpO_2_, skin temperature, and movement patterns, regardless of stress, will vary drastically across individuals, limiting the performance of general-purpose ML models.

To train ML models that predict BP based on a user’s wearable biometrics, we will develop and evaluate a series of both long short-term memory and transformer neural networks. The inputs to the models will consist of a separate 1D convolutional backbone for each biometric modality. The convolutional features will be fused upstream into a shared joint dense representation space and finally a dense prediction layer with linear activation for regression prediction. We will implement all models using Tensorflow (Google Brain) [15].

We will perform a series of self-supervised pretraining tasks to allow the networks to learn the baseline temporal dynamics of each individual’s biosignals. As a pretraining task, we will develop contrastive learning methods to automatically learn embeddings that encode the structure of the signal. For each wearable sensor modality, we will run a sliding window to isolate short-time segments. We will apply signal-based data augmentation techniques to derive a new signal. We will perform contrastive learning to learn neural network embeddings that maximize the similarity between each original segment and its modified version while minimizing the similarity across segments.

We will develop a modified version of the SimCLR (simple framework for contrastive learning of visual representations) algorithm, which will be tuned for the task of personalization to a user’s wearable signal readings. It is often the case that biosignals look highly similar, either due to temporal locality or relative homogeneity of the individual’s activity. To account for this possibility of recurring signal patterns, we will weigh the attract and repel strength of SimCLR based on the temporal distance between two segments of a particular signal. We will run a grid search to tune this repel strength.

The data augmentation techniques that we apply to the signals will be domain-specific, keeping in mind the inherent nature of each sensor. For example, for accelerometer data, rotations simulate different sensor placements and cropping is used to diminish the dependency of event locations [16]. Across several modalities, sensor noise can be simulated through scaling, magnitude-warping, and jittering [16]. We will be careful to not apply augmentation strategies that might change the meaning of the underlying signal.

As another pretraining task, we will perform generative pretraining by masking the input signal and predicting the missing portion of the signal using a deep autoencoder architecture. Pretraining in this manner will teach the model to understand the dynamics of each time series signal independent of BP or any other labels.

We will train the model on the first 60% of data (by time), tune hyperparameters on the next 20% of data, and calculate the mean absolute error and mean squared error on the final 20%. This evaluation pattern mimics real-world use, where a model will be calibrated by a user prior to real-world deployment. It is important to emphasize that we will train and test a separate personalized ML model for each individual.

We will evaluate the models by comparing the performance with respect to the number of labeled examples used for supervised fine-tuning. A plot of this comparison will elucidate the number of BP measurements required for model calibration to a single individual. We will plot the mean squared error at 10, 20, 30, 40, 50, 75, 100, 125, and 150 BP annotations, as these are feasible amounts of labels that might be provided by a user in real-world use. To ensure a robust evaluation, we will bootstrap at least 20 random samples of BP annotation subsets for each point on the x-axis and will report the mean and 90% CI. Just as in the plain supervised learning condition, we will create a separate plot for each study participant, as the ML portion of this proposal is testing the personalization of ML models rather than a general-purpose one-size-fits-all ML model which is more typical in ML evaluations.

We will perform a similar style of analysis for other clinical outcomes using publicly available data sets such as the Wearable Stress and Affect Detection (WESAD) [17] data set, a multimodal sensor data set for stress detection of nurses in a hospital [18], and K-EmoCon, a multimodal sensor data set for continuous emotion recognition in naturalistic conversations [19]. Each of these data sets, as well as several other publicly available data sets, contains several hours of multimodal biosignal data that overlap with the signals that we propose to collect, such as skin temperature, accelerometer streams, and HR. These data sets also include time-stamped annotations of end points that are likely to be correlated with BP, including self-perceived stress.

In prior work by other researchers, SSL pretraining approaches have repeatedly demonstrated improved performance over pure supervised learning in a variety of contexts [20-23]. Our preliminary data (see Results section) support that self-supervised pretraining on data solely from each individual results in improved models over purely supervised learning. While unlikely given our preliminary data and prior SSL publications, it is possible that minimal performance gains will be observed when applying the SSL strategies in a personalized manner. In such cases, the negative result would be a noteworthy finding due to prior successes of SSL.

## Results

We have developed a smartphone app, CardioMate, that will prompt participants to measure their BP and log their stress (Figure 2). The app comprises 2 primary screens, account and home. The account screen features user details, a star reward system for active participation in the study, and options to link 2 wearable devices (Fitbit and Omron Heartguide) for data synchronization with our secure, encrypted database. The home screen is divided into 6 sections, including questionnaires, messages, feedback, records, BP readings, and app instructions. Additionally, the CardioMate app includes an administrative area for study managers to view participant statistics and initiate personalized chats, complete with alarm and notification functions.

Data collection commenced on February 15, 2024. As of the manuscript submission date of February 24, 2024, a total of 2 participants have been recruited. The data collection period for each participant spans 28 days. Upon completion of the data collection period for each participant, we will proceed with the personalized machine learning model development to predict stress-induced BP spikes in real time. We aim to recruit a total of at least 45 participants and complete the relevant data collection, data analysis, and personalized ML development for each participant by the end of December 2024.

Our initial sets of published experiments have demonstrated promise for personalized SSL of stress but with some caveats. Our experiments on the WESAD data set demonstrated that deep learning model performance improves drastically when using self-supervised personalization when compared to personalization without SSL when there are a small number of labeled data points for supervision [24]. This effect diminishes with increasing amounts of labeled data [25,26], aligning with prior work that demonstrates that SSL is only beneficial under low-label settings. We have also tried these methods on a particularly challenging data set, a multimodal sensor data set for stress detection of nurses in a hospital [18]. This data set consists of wearable biosignals measured from nurses who wore Empatica E4 wristbands while conducting their normal shifts. This data set is difficult because (1) the data were collected in the wild rather than in controlled laboratory settings and (2) individual nurses were not consistent about their labeling practices, leading to sparse, irregular, noisy, and otherwise messy labels. Consequently, we found that the difference in area under curve and the receiver operating characteristic curve scores for self-supervised models was only about 2.5% higher on average compared against an equivalent baseline model [27], and this increase is within the margin of error due to the limited sample size. This lack of improvement in noisy annotation settings highlights the need for HCI innovations to improve data labeling quality for personalized AI within naturalistic settings.

We have also observed improved performance when personalizing affect-related prediction tasks without personalization both using classical ML [28] and deep learning [29], as well as when only applying SSL without personalization [30]. When disentangling and comparing the effects of SSL and personalization separately, we find that SSL yields more benefit than individualization on nonaffective medical data with large time intervals between data points, suggesting that the sampling frequency and other data considerations must be considered [30]. Collectively, these preliminary results demonstrate promise for the core ML approach that we propose.

## Discussion

The primary objective of this study is to leverage ML algorithms for real-time predictions of stress-induced BP spikes using consumer wearable devices such as Fitbit, providing actionable insights to both patients and clinicians to improve diagnostics and enable proactive health monitoring. Our study is motivated by recent research conducted at the John A Burns School of Medicine, which found that Native Hawaiian and other Pacific Islander individuals under a physician’s care for hypertension experienced an average drop of 18.3 points in systolic BP after participating in a 12-week hula program [2,3]. This study provides strong evidence that stress-reducing interventions can reduce hypertension in Native Hawaiian individuals. We hope to build upon this foundational research by leveraging consumer devices, such as Fitbit, to detect moments of high stress and provide just-in-time interventions that are culturally grounded. The first phase of this long-term research plan involves developing the AI necessary to power the digital intervention, which is the primary focus of this proposal.

The successful development of ML algorithms tailored to individual participants signifies a significant advancement in personalized health care interventions. By using longitudinal data from Fitbit devices and corresponding stress level labels from Ecological Momentary Assessment reports, the study will be able to capture individual-specific patterns effectively, enabling accurate predictions of stress-induced BP spikes. This approach not only enhances the understanding of stress-related hypertension but also provides opportunities for targeted interventions and improved patient outcomes.

Furthermore, the findings of this study contribute to the growing body of literature on the use of wearable devices and ML in health care. The adoption of transformer models for personalized prediction tasks, coupled with SSL techniques for pretraining, represents a novel approach to leveraging advanced computational techniques for real-time health monitoring. By dynamically weighing the importance of different time steps and focusing on relevant temporal features and dependencies, transformer models offer a powerful tool for predicting complex physiological responses such as stress-induced BP spikes. These findings will add to the existing literature by highlighting the potential of ML in improving the accuracy and efficiency of health monitoring systems, particularly in the context of personalized interventions for stress-related hypertension.

It is essential to acknowledge the limitations of this study design. One limitation is the relatively small sample size, which may limit the generalizability of the findings. Additionally, the study focuses primarily on predicting stress-induced BP spikes using wearable device data streams and may not capture other factors contributing to hypertension. Future research should aim to address these limitations by including larger and more diverse samples and exploring additional predictors of hypertension.

The findings of this study will demonstrate the feasibility and potential benefits of leveraging ML algorithms for real-time predictions of stress-induced BP spikes using consumer wearable devices. By developing personalized AI models based on individual biosignals, the study will provide valuable insights into the monitoring and management of stress-related hypertension. These findings will have broader implications for personalized health care interventions and underscore the importance of integrating advanced computational techniques into health care systems to improve patient outcomes. Through iterative refinement and optimization, we aim to develop a personalized deep-learning framework capable of accurately predicting stress-induced BP spikes, thereby promoting individual well-being and health outcomes.

## Appendix

Multimedia Appendix 1 Peer-review reports from the Medical Research Advisory Committee of the Hawaii Community Foundation (HCF).

## Abbreviations

AI: artificial intelligence
AMIA: American Medical Informatics Association
API: application programming interface
AWS: Amazon Web Services
BP: blood pressure
CHI: Computer-Human Interaction
HIPAA: Health Insurance Portability and Accountability Act
HR: heart rate
IEEE: Institute of Electrical and Electronics Engineers
ML: machine learning
NIH: National Institutes of Health
PSB: Pacific Symposium on Biocomputing
PSS: Perceived Stress Scale
SimCLR: simple framework for contrastive learning of visual representations
SpO_2_: blood oxygen level
SSL: self-supervised learning
WESAD: Wearable Stress and Affect Detection

## Data Availability

Upon the completion of the data collection phase, the data sets generated and analyzed during this study will be available in a public repository. The data citation should include a persistent identifier (eg, web URL or DOI) cited per journal style in the reference list. The amassed data will be fully anonymized and made accessible to the academic community. Subsequently, comprehensive guidelines, together with a designated link facilitating data retrieval and use, will be provided.
