# Supplementary material for: Personalized AI-Driven Real-Time Models to Predict Stress-Induced Blood Pressure Spikes Using Wearable Devices: Proposal for a Prospective Cohort Study
Source: JMIR Res Protoc. 2024 Mar 25;13:e55615. doi: 10.2196/55615 (PMC11002732; doi:10.2196/55615)
Supplement: Multimedia Appendix 1 [file resprot_v13i1e55615_app1.pdf]

HAWAIʻI COMMUNITY FOUNDATION

June 01, 2023

Vassilis Syrmos
Interim Director
UNIVERSITY OF HAWAII - OFFICE OF RESEARCH SERVICES
2440 Campus Rd., Box 368
Honolulu, HI 96822-2234

Aloha,

On behalf of the Medical Research Advisory Committee of the Hawaiʻi Community Foundation (HCF), I am pleased to inform you that a grant has been awarded to your organization. We are excited about your project and believe it is the kind of work that can help to make a difference in Hawaiʻi.

Grant ID#: MedRes_2023_00002689 **Grant Amount:** $59,946.84
Fund: Robert C. Perry Fund;
Grant Purpose: Personalized Prediction of Blood Pressure Spikes in Real Time using Wearable and Smartphone Data
Principle Investigator: Peter Washington
Grant Period: 06/01/2023 - 12/01/2024
Final Report: Due one month after the grant end date
Grant Conditions: Payment is contingent upon IRB approval submission.

The advisory committee recommends that you reach out to a cardiologist with research experience for additional mentorship. One specific recommendation was Dr. Andras Bratincsak who is doing AI work using EKGs.

Please read the following grant terms carefully:

Purpose: This grant is to be used only for the purpose designated. If there is a change in the nature of the activities described in the proposal for which these funds were granted, please contact HCF before expending the funds.

Reporting: A required grant activities and financial report is due to HCF 1 month after the grant end date. Contact HCF staff if you cannot submit the report by this date.

Site Visit: HCF reserves the right to follow up this grant with a site visit while the project is in progress or once the project has been completed. You might be asked to provide HCF with a brief interim report indicating the progress of the project.

Unexpended Funds: You must notify HCF of any unexpended funds at the end of the grant period, or if (i) your organization has not performed in accordance with this agreement, (ii) your organization loses its exemption from federal income taxes under Section 501(c)(3) of the Internal Revenue Code, or (iii) your organization materially

changes its programs, activities or mission.

Publicity: Please review the attached *Guide to External Announcements for Grantees* for information on publicity of the grant award. For more information on publicizing your HCF grant, please contact Chips DaMate at cdamate@hcf-hawaii.org.

At this time, HCF is transitioning to electronic correspondence and a check is to follow in the mail. By cashing the referenced check, you thereby accept and agree to follow the above grant terms.

Enclosed are comments from the reviewers pertaining to the submitted proposal. Should you have any questions call me at (808) 566-5523 or email at tmabellos@hcf-hawaii.org. I wish you great success and look forward to hearing about your project.

Here is the Payment and Conditions Schedule for your grant.

Final Schedule

	Type	Report Type	Scheduled Date	Amount	Notes
1	**Payment**		06/01/2023	$59,946.84	
2	**Conditions**		11/30/2023		Payment is contingent upon IRB approval submission.
3	**Report Upload Only**	**Final Report**	01/01/2025		
			**Payment Total**	**$59,946.84**	

Sincerely,

Tricia Mabellos

Program Officer, Community Grants & Initiatives

Hawaii Community Foundation
Medical Research Program
Proposal Review
May 24, 2023

**Project Title:**	Personalized Prediction of Blood Pressure Spikes in Real Time from FitBit Data using Artificial Intelligence
**Applicant Organization:**	University of Hawaii - Office of Research Services
**Department:**	Information and Computer Sciences
**Principal Investigator:**	Peter Washington
**Amount Requested:**	$59,946.84
**Request ID:**	MedRes_2023_00002689
**Area submitted to:**	Clinical Research - Cancer, Heart Disease, or Lung Disease;Clinical Research - General

	Reviewer 1	Reviewer 2	Average	Final Avg
**Score**	2	3	2.5	2.40

Reviewer 1 Comments:

Overview

Overall, this is an exciting project from an ESI/new investigator recently recruited to UH Manoa, who is developing machine learning tools for personalized analysis/prediction of blood pressure spikes using commercial wearable health monitors (FitBit and Omron). This is a small-scale clinical study on a representative cross-section of Hawaii patients that will lay a solid scientific foundation, by developing new algorithms and gathering preliminary data, for larger follow-up studies on hypertension in Hawaii's populations. The PI is outstanding, has strong support as a new UHM faculty, and has found an exceptional mentor for this project. The approach is rigorous but feasible in the timeframe of this HCF award. There were some minor weaknesses, including inconsistency of patient number in the study and unclear amount of effort for the PI, but overall, a very well-written proposal for an exceptional project.

Research plan and clarity of specific aims

The goal of this proposal is to apply machine learning techniques to analyze and integrate individual patient data collected over 4-weeks from two different wearable health monitoring devices (FitBit and Omron). The personalized self-supervised learning methods will be trained to predict high blood pressure spikes using the biosignal data, with the ultimate goal of building foundational AI tools for advanced personalized medicine. The proposal is very well-written and clear in its objectives and methodology. The grant is organized in three Specific Aims: 1) Create a novel dataset of wearable sensor data and corresponding blood pressure measurements. 2) Develop a personalized self-supervised pre-training procedure for time series data using both contrastive learning and masked predictions. 3) Develop a novel personalized pre-training procedure which exploits the multimodal nature of wearable time series data. This is a clinical study with the goal of recruiting 40 patients (ages 30-70, representative of Hawaii's demographics) from the PI's network of collaborators. The PI has been building expertise in developing new machine learning approaches for analyzing longitudinal datasets from single individuals, versus pooling data from multiple patients (with poorer personal

annotation). The PI has also already established an impressive network of clinical collaborators, and a good laboratory team of students. The PI did an excellent job of discussing potential pitfalls and alternative approaches, with realistic plans to meet recruitment and retention challenges. Couple of concerns, although mostly minor. First, the main proposal says they intend to recruit 40 patients, but the budget section says 60 patients (assume it's a typo). Second, there is a concern about the recruiting strategy here, using the PI's collaborations with the Department of Psychiatry. In the long run, the PI should consider collaborations with primary care physicians (or cardiologists etc), as recruitment from a pool of patients receiving inpatient or outpatient psychiatric services may introduce confounding or recruitment bias. However, as a preliminary study, these weaknesses are relatively minor (but worth noting).
**Significance**<br>New approaches to fight hypertension and cardiovascular diseases are of high public health significance, and using novel machine learning approaches for personalized medicine is highly innovative. This project is exciting and with high potential impact. Moreover, it will likely lay a critical foundation for larger clinical studies by this PI and his team. There is good preliminary data from the PI that supports the feasibility of this project.
**PI qualifications**<br>This proposal is from a new investigator (tenure-track Assistant Professor) in UH Manoa's Department of Information & Computer Sciences. The PI recently graduating from Stanford in 2022 with a PhD in Bioengineering and also received a Masters from Stanford in Computer Science. Since 2022, he has been establishing a research program in machine learning for health and precision medicine, and building exciting interdisciplinary collaborations in Hawaii. He has a strong track record of productivity and is well-supported by his Chair and department. The mentor for this project, Dr. Kaholokula, is well-experienced in clinical studies of blood pressure in Hawaii and will certainly be a fantastic resource (and potential collaborator) for this new investigator.
**Appropriateness of budget**<br>Overall, the budget is well-planned and justified. The major proportion of the budget is to support a graduate research assistant working on this project, which is appropriate. The other costs are all reasonable, and the project is also supported in-kind by the PI's startup funds. The budget includes no salary support for the PI (in-kind effort), however one concern is that the PI does not specify the % effort he will devote to this particular project. He should be listed in the Personnel section with % effort, even if he is not requesting salary support.
**Other considerations including preference to proposals by recently qualified researchers, and relevance to Hawaiʻi**<br>The PI is a recently qualified researcher, having started as a new tenure-track Assistant Professor at UH Manoa in 2022. Furthermore, support of new PIs with expertise in data science are high priorities for Hawaii biomedical research community. This proposal does have specific relevance to Hawaii since it studies hypertension in a local patient population, and aims to create personalized machine learning tools based on these Hawaii patients. Native Hawaiians and Other Pacific Islanders have a significantly higher rate of mortality due to heart disease.
**Other Support**<br>There is no apparent scientific overlap with the PI's other proposals.

Additional Reviewer's Comments
None.

Reviewer 2 Comments:

Overview
In this project PI aims to develop a strategy to predict blood pressure spikes. This strategy is based on the development of a computer algorithm, or artificial intelligence (AI), based on the data acquired from simultaneous use of Fitbit and Omron HeartGuide wearable devices. Fitbit acquires data on heart rate, blood oxygen level, breathing rate, gyroscope, and skin temperature while Omron measures blood pressure. The combined data from two devices can be used to develop AI. The proposal includes all necessary components but could have been prepared better. Some portions of the write up are hard to follow, and not enough emphasis is placed on description of significance of the project.

Research plan and clarity of specific aims
Three aims are proposed. Aim 1 regarding data collection is well presented. Forty participants (5 at a time) will be simultaneously wearing Omron and Fitbit for 4 weeks (at least 15 hrs/day), allowing for data collection. The recruitment and exclusion plans are well described, with attention paid to group diversity. It is also well explained how participants will be monitored and contacted during the study. The obtained data will be novel and important. This aim is considered strong. The remaining two aims are difficult to assess due to the way they are presented. The entire description of data analysis is described using specialized jargon, in a manner not understandable to non-expert. Preliminary Data do not seem to be too closely relevant to this proposal and seem to have been gathered as part of a different research project; it is also poorly presented. The project is collaborative, with PI (an expert in computer science and AI) working with investigator from the medical school.

A minor weakness of the approach is that it is based on use of five participants wearing 5 device pairs at a given time. There is no mention what happens if one or more devices are lost or damaged.

Significance
This is the weakest part of this proposal. PI states that the newly developed AI will allow for real time prediction of blood pressure spikes, and that this can be used to alert patients about undesirable activities and adverse behaviors that can lead to these spikes, and to assess how frequent they are. But further description how can this leveraged to improve health is not included. It would be more valuable if PI focused on explaining this aspect of work, rather than providing 2 pages of indigestible to non-expert description of data analysis.

PI qualifications
PI is well qualified. He is publishing well and his productivity with grant submissions is admirable. In addition to the grants submitted as a PI, he serves as a co-investigator on several pending grants from JABSOM which attests to an interest in his expertise and skill set leading to interdisciplinary collaborations.

Appropriateness of budget
Budget is appropriate. Matching funds are described. Most of the funds will be used to support a GA so a training component is included.

Other considerations including preference to proposals by recently qualified researchers, and relevance to Hawaiʻi
PI is a recently qualified researcher, in an Assistant Professor position. He has been well mentored thus far and will continue to be mentored during this project. Hypertension and high blood pressure are commonly experienced by Native Hawaiians, Pacific Islanders, Filipinos, and other ethnic groups of Hawaii so the project is relevant to Hawaii.

Other Support
PI is supported by start-up funds and will use them to provide matching funds for this project.

Additional Reviewer's Comments
Two of the pending grants seem to be similar to this application, Although PI is making a case on why there is no overlap, it is rather clear that almost the entire approach is the same – the difference is in a focus on a different patient cohort and other medical problem. Parts of this proposal were taken verbatim from previous submission.

IRB has been submitted.
